# Unlocking hybrid vigor: a new genic male sterile gene *in da house*

**DOI:** 10.1093/plphys/kiaf523

**Published:** 2025-10-13

**Authors:** María Flores-Tornero, Chong Teng (滕冲)

**Affiliations:** Assistant Features Editor, Plant Physiology, American Society of Plant Biologists; Departament de Biologia Vegetal, Facultat de Ciències Biològiques, Universitat de València, Valencia 46100, Spain; Assistant Features Editor, Plant Physiology, American Society of Plant Biologists; Department of Plant Sciences and Genome Center, University of California, Davis, CA 95616, USA

Seed and fruit production, the foundation of the human food supply, relies on sexual reproduction in plants. In flowering plants, pollen grains produced in the anthers fertilize ovules, forming seeds that will be protected inside the fruits. Many plants preferentially self-pollinate (Irish 2017), but this natural process can be agronomically disadvantageous.

Many plant breeders prefer to cross-pollinate their plants to produce hybrids with an improved agronomic performance compared with their parents, a phenomenon called hybrid vigor (Groszmann et al. 2014). Those hybrids are typically obtained either by artificial interventions or natural mechanisms that prevent self-pollination and ensure cross-pollination. In many crops such as pepper and soybean, hybrids are produced by anther removal before pollen releases, but this is a time- and labor-consuming task.

Fortunately, several mutations that cause male sterility and enable hybrid seed production have been identified that are either naturally occurring or produced by gene editing (Chen et al. 2021; Zhang et al. 2023). To trigger male sterility, plant breeders originally used cytoplasmic male sterility; however, due to its lack of stability, breeders are shifting to the gene male sterility approach (GMS). In Arabidopsis, rice, and maize, GMS is already widely used, whereas in other species such as pepper, tomato, cucumber, and related vegetables, hybrid production still relies on time-consuming emasculation practices. In pepper, more than 20 loci controlling GMS have been reported, but only 3 male-sterile (ms) loci have been identified (Jeong et al. 2018; Cheng et al. 2020; Dong et al. 2022).

In this issue of *Plant Physiology*, Du et al. identified and characterized *Male sterile from China-4* (*Msc-4*), a new natural GMS allele in pepper, widening the possibilities for hybrid seed production.

The authors observed that the vegetative and reproductive growth of *msc-4* resembles the fertile version, but fruits are smaller and seedless. Although *msc-4* anthers have no pollen, the ovules are fertile, and cross-pollinations with fertile *Msc-4* pollen produces fertile progeny (Fig. 1). Moreover, authors confirmed that *msc-4* is a new independent allele by outcrossing with the other 3 already known *msc* lines. Furthermore, the authors confirmed the stability of the sterile phenotype regardless the location by growing *msc-4* mutants in different locations.

**Figure 1.**
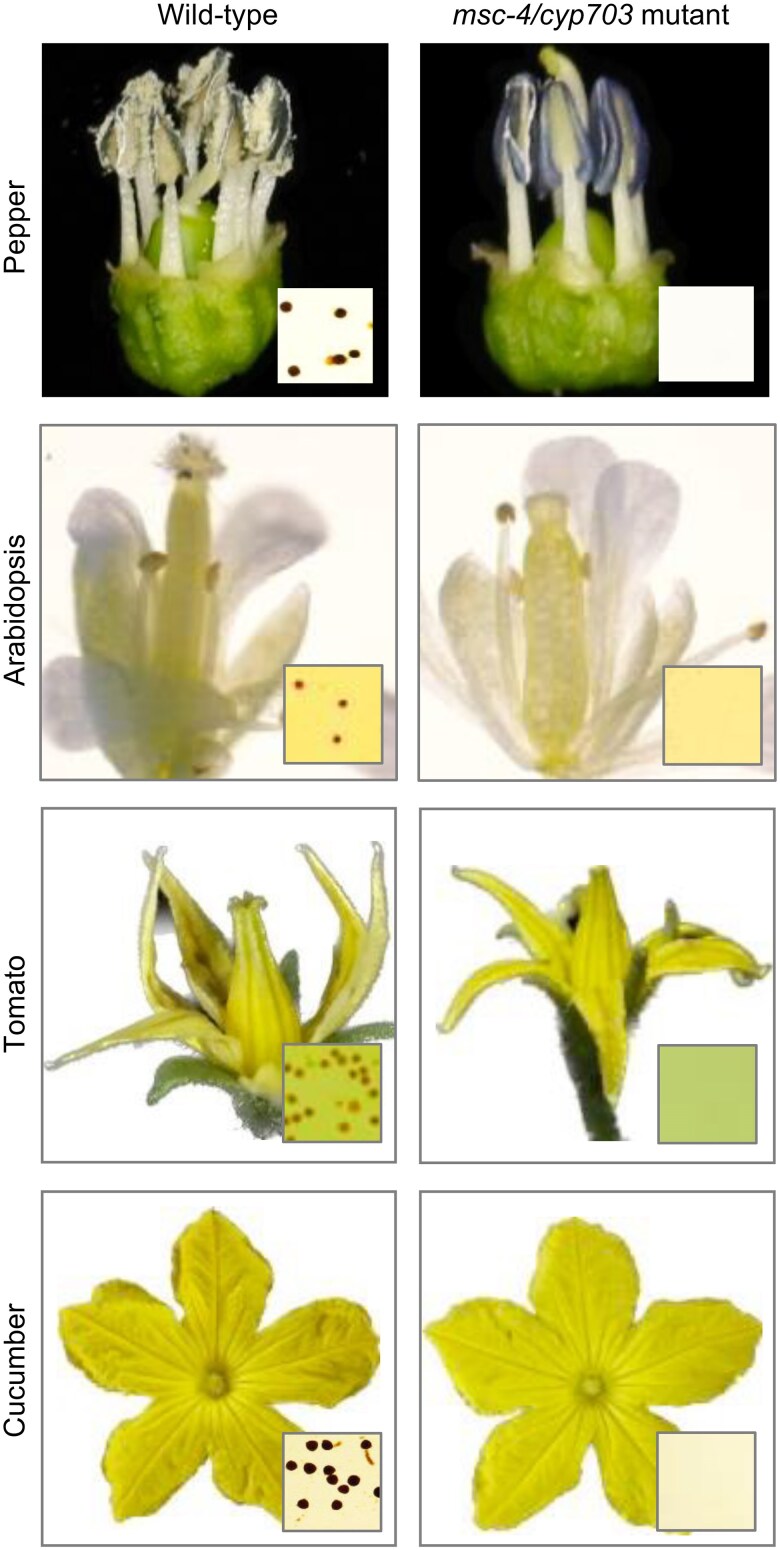
Comparative floral phenotypes of *msc-4/cyp703* mutants and wild-type plants in pepper, Arabidopsis, tomato, and cucumber. Insets show pollen grains (stained or absent) from the corresponding flowers. Figures were adapted from Du et al. 2025.

To further dissect the cause of the sterility problem, Du et al. characterized anther and pollen development of *msc-4* and wild type by using complementary microscopy techniques. At stage 5, Du et al. observed that tapetal cells, the outer layer of cells providing nutrients and waxy materials building the pollen wall of *msc-4*, were abnormally full of vacuoles and microspore formation was compromised, causing shrunken and collapsed microspores. These results indicate that male sterility is due to an abnormal tapetum and pollen wall formation.

To identify the gene corresponding to this defect, Du et al. used high-throughput short-read sequencing-based bulked segregation analysis and sequence variation calling to locate the *msc-4* locus. In the region of interest, 11 candidate proteins were annotated (open reading frame [ORF]1-ORF11), but only 4 were expressed in developing anthers. ORF1 was the best candidate for the *msc-4* locus because the authors identified in this region a point mutation that prematurely stops the protein, and silencing experiments of ORF1 yielded no pollen.

The ORF1 gene encodes CYP703A2, an ortholog of the Arabidopsis cytochrome P450 monooxygenase required for lipid metabolism during pollen wall formation and exine pollen development. It was previously shown that loss of *CYP703* function disrupts pollen exine development, resulting in complete male sterility in Arabidopsis (Morant et al. 2007).

To understand the molecular mechanisms that produce male sterility in *msc-4* peppers, transcriptomic sequencing was performed in anther samples at different developmental stages. After differentially expressed gene identification and clustering, the authors performed a gene enrichment analysis with differentially expressed genes from stage 5, relating them with pollen wall assembly and lipid metabolism.

In addition, phylogenetic analyses indicated the conservation of *Msc-4* across species, so the authors used *Msc-4* from pepper to complement the sterile phenotype of the Arabidopsis *cyp703a2*. Moreover, the CRISPR-generated knockout of *CYP703* in tomato and cucumber caused male sterility (Fig. 1), strongly supporting the similar role of MSC-4 and CYP703A2 as well as its high conservation.

In conclusion, a natural mutant of *CYP703* (*msc-4*) confers genic male sterility not only in pepper but also in tomato and cucumber because *Msc-4/CYP703* encodes an enzyme involved in lipid metabolism during pollen exine formation. Future research may expand our understanding of this mechanism in other crops, clarifying how lipid metabolism integrates with tapetal development and leading to new biotechnological tools to improve hybrid production.

## Data Availability

No new data were generated or analyzed in support of this research.
